# Diagnostic and clinical associations of serum Isthmin-1 in acute ischemic stroke

**DOI:** 10.1007/s11845-026-04383-2

**Published:** 2026-04-16

**Authors:** Hasan Hüseyin Sarkı, Melih Yüksel, Demet Yıldız, Kağan Huysal, Mehmet Oğuzhan Ay, Yeşim İşler, Halil Kaya, Umut Ocak, Muhammed Said Karaca, İsmail Ayan

**Affiliations:** 1 Department of Emergency Medicine, University of Health Sciences, Bursa Yuksek Ihtisas Training and Research Hospital, Bursa, Türkiye; 2 Department of Neurology, University of Health Sciences, Bursa Yuksek Ihtisas Training and Research Hospital, Bursa, Türkiye; 3 Department of Clinical Biochemistry, University of Health Sciences, Bursa Yuksek Ihtisas Training and Research Hospital, Bursa, Türkiye

**Keywords:** Ischemic stroke, Isthmin-1, Modified Rankin Scale, Mortality, Biomarker

## Abstract

**Background:**

This study aimed to evaluate the diagnostic value of serum Isthmin-1 levels and their associations with radiological severity, vascular territory involvement, and clinical outcomes in patients with acute ischemic stroke.

**Methods:**

This prospective, single-center study was conducted between January 1 and June 30, 2024. Serum Isthmin-1 levels were measured at admission. Clinical characteristics, radiological findings, and stroke-related scores were recorded. Radiological severity was assessed using the Alberta Stroke Program Early CT Score (ASPECTS), and functional outcomes were evaluated at 90 days using the modified Rankin Scale (mRS).

**Results:**

The median age of stroke patients was 71 years (IQR 63–79), and the median serum Isthmin-1 level was 393.72 ng/mL (IQR 326.34–477.22). Serum Isthmin-1 levels were significantly higher in patients with acute ischemic stroke compared with healthy controls (*p* < 0.001). Patients with anterior cerebral artery infarction had significantly lower Isthmin-1 levels than those with other vascular territory involvement (*p* = 0.005). A weak but significant positive correlation was observed between Isthmin-1 levels and ASPECTS scores (*r* = 0.252, *p* = 0.007). No significant association was found between Isthmin-1 levels and 90-day functional outcomes or early mortality.

**Conclusions:**

Serum Isthmin-1 levels are elevated in acute ischemic stroke and are associated with vascular territory involvement and radiological severity. Although not predictive of functional outcomes or early mortality, Isthmin-1 may reflect hypoxia-related endothelial and inflammatory processes and could serve as an adjunctive biomarker in the acute evaluation of ischemic stroke.

**Supplementary Information:**

The online version contains supplementary material available at https://doi.org/10.1007/s11845-026-04383-2.

## Introduction

Stroke is a major neurological disorder caused by the sudden interruption or reduction of cerebral blood flow and is associated with substantial mortality and long-term morbidity. Approximately 70–85% of all stroke cases are ischemic in origin. Globally, stroke ranks as the second leading cause of death and is considered one of the leading causes of permanent disability in the adult population. The effectiveness of acute treatment options, including intravenous thrombolysis and mechanical thrombectomy, largely depends on patients’ timely access to the healthcare system. However, the literature indicates that awareness of stroke symptoms remains insufficient in many communities, leading to delays in healthcare-seeking behavior following symptom onset. Such delays result in missed therapeutic windows, increased mortality rates, and severe long-term neurological sequelae [1].

Early risk stratification plays a pivotal role in reducing morbidity and mortality in patients with acute ischemic stroke. Several clinical and radiological assessment tools—such as the National Institutes of Health Stroke Scale (NIHSS), Alberta Stroke Program Early CT Score (ASPECTS), Bamford classification, and the modified Rankin Scale (mRS)—are widely used to assess disease severity, estimate prognosis, and guide therapeutic decision-making [2–5]. Although these tools are well established, they primarily reflect clinical presentation and imaging findings and may not fully capture the underlying molecular and pathophysiological processes driving ischemic brain injury.

Isthmin-1 is a glycoprotein originally identified in embryonic brain tissue that contains AMOP and thrombospondin type 1 repeat (TSR)-like domains and is expressed in a wide range of tissues. Emerging evidence indicates that Isthmin-1 plays a regulatory role in metabolic homeostasis, inflammatory signaling, endothelial function, and vascular integrity [6]. Importantly, the upregulation of Isthmin-1 expression via hypoxia-inducible factor-1 (HIF-1)–mediated transcription suggests that this molecule may be biologically active during conditions of tissue hypoperfusion and ischemia [7]. Furthermore, reported associations between serum Isthmin-1 levels and obesity, insulin resistance, and lipid metabolism point to a broader involvement in metabolic and vascular dysfunction [8]. Given the central roles of hypoxia, endothelial injury, and inflammation in the pathophysiology of ischemic stroke, Isthmin-1 represents a biologically plausible candidate biomarker in ischemic cerebrovascular disease.

Therefore, the aim of this study was to evaluate the diagnostic and prognostic significance of serum Isthmin-1 levels in patients with acute ischemic stroke and to investigate its association with radiological severity, vascular territory involvement, and clinical outcomes.

## Materials and methods

### Study design and ethical approval

This hospital-based, single-center, prospective study was conducted in the Department of Emergency Medicine at Bursa Yüksek İhtisas Trainingand Research Hospital, University of Health Sciences. Prior to study initiation, ethical approval was obtained from the Clinical Research Ethics Committee of Bursa Yüksek İhtisas Training and Research Hospital (Approval No: 2011-KAEK-25; Decision No: 2023/11–04).

## Study population

Participants who presented to the emergency department of our hospital between January 1, 2024, and June 30, 2024, and who agreed to participate in the study were enrolled. The participants were divided into two groups:

- Patient Group: Patients who presented to the emergency department for various reasons and were diagnosed with acute ischemic stroke following clinical, laboratory, and radiological evaluations.
- Control Group: Healthy volunteers aged ≥ 18 years with no previous history of cerebrovascular disease.

Written informed consent was obtained from all participants or their first-degree relatives prior to enrollment.

## Inclusion criteria

Patient Group:

1. Age ≥ 18 years.
2. Diagnosis of ischemic stroke.
3. Provision of written informed consent by the participant or a first-degree relative.

Control Group:

1. Age ≥ 18 years.
2. No prior history of cerebrovascular disease.
3. Provision of informed consent.

## Exclusion criteria

Patient Group:

1. Age < 18 years. 
2. Absence of informed consent
3. Hemorrhagic stroke
4. Transient ischemic attack
5. Presence of a cranial mass lesion
6. Previous history of cerebrovascular disease

Control Group:

1. Age < 18 years.
2. Refusal to provide informed consent.
3. Previous history of cerebrovascular disease.

## Data collection and laboratory methods

For patients in the study group, presenting complaints, vital signs, comorbidities, medical history, laboratory findings, radiological imaging results, electrocardiography findings, and emergency department outcomes (discharge, ward admission, intensive care unit admission, death, or refusal of treatment) were systematically recorded. In patients with acute ischemic stroke, venous blood samples were obtained after informed consent. Blood samples were obtained within the first 10 min of emergency department admission. The time from symptom onset to hospital admission was recorded for each patient (median: 7 h, IQR: 4–16.25 h). A total of 3 mL of venous blood was collected into serum separator tubes. Samples were centrifuged at 4000 rpm for 10 min, and serum was stored at − 80 °C until analysis. The same protocol was applied to the control group.

Serum Isthmin-1 levels were measured in all samples using the enzyme-linked immunosorbent assay (ELISA) method. A commercially available SunRed^®^ ELISA kit (Shanghai Sunred Biological Technology, China) was used for analysis. Optical density measurements were performed at a wavelength of 450 nm using a ChroMate 4300 microplate reader (Awareness Technology Inc., USA).

A total of 212 participants were included in the study, comprising 114 patients in the ischemic stroke group and 98 healthy individuals in the control group.

### Follow-up and outcomes

Mortality status and modified Rankin Scale (mRS) scores were assessed through telephone interviews with participants and/or their relatives or by reviewing hospital records via the Hospital Information Management System.

- Primary Outcome:. Association between serum Isthmin-1 levels and early prognosis, including 24-hour mortality. Due to the limited number of early mortality events, this outcome was analyzed descriptively and interpreted with caution.
- Secondary Outcome: To evaluate the relationship between serum Isthmin-1 levels and functional outcome as assessed by the modified Rankin Scale at 90 days.

### Statistical analysis

Statistical analyses were performed using IBM SPSS Statistics for Windows, Version 27.0 (IBM Corp., Armonk, NY, USA). Descriptive statistics were expressed as mean ± standard deviation, median with range and/or interquartile range (IQR) for continuous variables, and as frequency and percentage for categorical variables.

The Kolmogorov–Smirnov test was used to assess normality of data distribution, and Levene’s test was applied to evaluate homogeneity of variances. For continuous variables that did not meet parametric test assumptions, differences between groups were analyzed using the Kruskal–Wallis and Mann–Whitney U tests. Spearman correlation analysis was employed for non-parametric variables. Results were reported with a 95% confidence interval, and a p-value < 0.05 was considered statistically significant.

## Results

A total of 212 participants meeting the inclusion criteria were enrolled, including 114 patients with acute ischemic stroke and 98 individuals in the control group. The median age was 71 years (IQR 63–79) in the stroke group and 51.5 years (IQR 43–60) in the control group. Male sex was observed in 65 patients (57.0%) and 53 controls (54.1%). Among stroke patients, the most common presenting symptom was paresis or paresthesia (*n* = 72, 63.2%). At least one comorbidity was present in 83 patients (72.8%), with hypertension being the most frequent (*n* = 69, 60.5%). Regular medication use was reported by 83 patients (72.8%), most commonly antihypertensive agents (*n* = 63, 53.5%). Atrial fibrillation was detected on electrocardiography in 17 patients (14.9%). Neuroimaging revealed anterior circulation infarction in 59 patients (51.8%), most frequently involving the middle cerebral artery (MCA) territory (*n* = 53, 46.5%). Mechanical thrombectomy was performed in 10 patients (8.8%), and intravenous thrombolytic therapy was administered to 2 patients (1.8%). At 90-day follow-up, an mRS score of 1 was observed in 41 patients (36.0%) (Table 1).

**Table 1:** Clinical and Demographic Information

Patient Group Age (years)^*^		71 (63–79)
Control Group Age (years)^*^		51.5 (43–60)
Patient Group Gender^#^	Male	65 (57.0)
	Female	49 (43.0)
Control Group Gender^#^	Male	53 (54.1)
	Female	45 (45.9)
Application Complaints ^#^	Paresis/Paresthesia	72 (62.2)
	Dysarthria	59 (51.8)
	Vertigo	41 (36.0)
	Plegia	25 (21.9)
	Aphasia	22 (19.3)
	Altered consciousness	16 (14.0)
	Other	12 (10.5)
Alcohol Use ^#^		0
Tobacco Use ^#^		2 (1.8)
History of TIA^#^		1 (0.9)
Comorbidities^#^		83 (72.8)
Comorbidities^#^	Hypertension	69 (60.5)
	Diabetes Mellitus	39 (34.2)
	Hyperlipidaemia	25 (21.9)
	Chronic Renal Failure	5 (4.4)
	Other	27 (19.6)
Heart Failure ^#^		20 (17.5)
Atrial Fibrillation^#^		17 (14.9)
Medication Use ^#^		83 (72.8)
Medication Use ^#^	Antihypertensive	61 (53.5)
	Antiaggregan	38 (33.3)
	Oral Antidiabetic Agent	26 (22.8)
	Beta Blocker	20 (17.5)
	Calcium Channel Blocker	12 (10.5)
	Anticoagulant	13 (11.4)
	Antilipidemic	13 (11.4)
	Insulin	4 (3.5)
	Other	5 (4.4)
System Region ^#^	Anterior System	59 (51.8)
	Posterior System	50 (43.9)
	Anterior+Posterior System	5 (4.4)
Ischemic Region^#^	Middle cerebral artery	53 (46.5)
	Posterior cerebellar artery	46 (40.4)
	Lacunar	30 (26.3)
	Anterior cerebral artery	6 (5.3)
	Basilar artery	7 (6.1)
	Superior cerebellar artery	4 (3.5)
	Posterior inferior cerebellar artery	7 (6.1)
	Internal carotid artery	9 (7.9)
	Multiple vessels	8 (7.0)
BAMFORD Classification^#^	Posterior circulation infarction	46 (40.4)
	Partial anterior circulation infarction	45 (39.5)
	Lacunar infarction	23 (20.2)
Emergency Department Outcome^#^	Service admission	89 (78.1)
	Admission to stroke/intensive care unit	22 (19.3)
	Other	3 (2.7)
Thrombolytic^#^		2 (1.8)
Mechanical Thrombectomy^#^		10 (8.8)
Thrombolytic+Mechanical Thrombectomy^#^		0
90-day modified Rankin scale^#^	0	13 (11.4)
	1	41 (36.0)
	2	19 (16.7)
	3	14 (12.3)
	4	12 (10.5)
	5	6 (5.3)
	6	9 (7.9)
Mortality within 24 h		1 (0.9)
Mortality within 90 days		9 (7.9)
Total^#^		114 (100)

^#^ n (%), ^*^ Median (IQR 25–75), TIA; Transient Ischemic Attack

Median serum Isthmin-1 levels were significantly higher in the stroke group compared with controls (393.72 ng/mL [IQR 326.34–477.22] vs. 209.64 ng/mL [IQR 155.27–271.33], *p* < 0.001).

The median admission NIHSS score was 3 (IQR 1–5), and the median ASPECTS score was 10 (IQR 9–10). Mean systolic blood pressure was 156.1 ± 24.6 mmHg, median heart rate was 70 beats/min (IQR 65–78), and mean hemoglobin level was 13.1 ± 1.9 g/dL (Table 2).

**Table 2:** Clinical and Laboratory Data of Patients

Variables	Value
Time to onset of symptoms, hours Median IQR (25–75)	7 (4-16.25)
Baseline NIHSS score, Median IQR (25–75)	3 (1–5)
ASPECT score, Median IQR (25–75)	10 (9–10)
90-day mRS, Median IQR (25–75)	2 (1–3)
GCS, Median IQR (25–75)	15 (15–15)
Pulse rate/min, Median IQR (25–75)	70 (65–78)
SBP mm/Hg, Mean ± SD	156.1 ± 24.6
DBP mm/Hg, Median IQR (25–75)	88.5 (78–100)
MAP mm/Hg, Mean ± SD	110.5 ± 17.0
Respiratory rate/min, Median IQR (25–75)	14 (13–15)
Oxygen saturation, Median IQR (25–75)	99 (99–99)
Haemoglobin, g/dL Mean ± SD	13.1 ± 1.9
Haematocrit, Mean ± SD	39.4 ± 5.3
MCV, fL Median IQR (25–75)	88 (84.1–91.3)
Leukocyte count, 10^3/mL Median IQR (25–75)	8.89 (7.4-10.85)
Neutrophil count, 10^3/mL Median IQR (25–75)	6.2 (4.6–7.8)
Lymphocyte count, 10^3/mL Median I (25–75)	1.9 (1.5–2.5)
Platelet count, 10^3/mL Median IQR (25–75)	225 (190.75–277)
MPV, fL Median IQR (25–75)	10.8 (10-11.9)
BUN, mg/dL Median IQR (25–75)	20 (16–25)
Creatinine, mg/dL Median IQR (25–75)	0.9 (0.74–1.10)
Potassium, mEq/L Median IQR (25–75)	4.4 (4.0-4.8)
Sodium, mEq/L Median IQR (25–75)	138 (137–140)
ALT, U/L Median IQR (25–75)	16 (12.75-22)
AST, U/L. Median IQR (25–75)	24 (19–32)

mRS; Modified Rankin Scale, GCS; Glasgow Coma Scale, SBP; Systolic Blood Pressure, DBP, Diastolic Blood Pressure, MAP; Mean Arterial Pressure, MCV; Mean Corpuscular Volume, MPV; Mean Platelet Volume, BUN; Blood Urea Nitrogen, ALT; Alanine Aminotransferase, AST; Aspartate Aminotransferase

Serum Isthmin-1 levels were significantly higher in patients presenting with visual disturbance or diplopia compared with those without these symptoms (*p* < 0.05), whereas no significant difference was observed according to sex (Table 3). Analysis by vascular territory demonstrated significantly lower Isthmin-1 levels in patients with anterior cerebral artery (ACA) territory infarction compared with other territories (*p* = 0.005) (Table 4).

**Table 3:** Analysis table of variables with Isthmin-1

Variables		*N*	Isthmin-1	*p*-value *
Gender	Male	65	394.83 (345.16-484.36)	> 0.05
	Female	49	390.39 (305.57-457.18)	
Plegia	No	89	392.61 (326.57–470.20)	> 0.05
	Yes	25	402.67 (299.04-512.81)	
Paresis/Paresthesia	No	42	392.01 (315.12-467.96)	> 0.05
	Yes	72	397.03 (326.51–482.80)	
Dysarthria	No	55	379.41 (326.49-467.84)	> 0.05
	Yes	59	397.06 (317.94-483.75)	
Aphasia	No	92	378.15 (318.83-470.86)	> 0.05
	Yes	22	418.65 (370.68-624.47)	
Altered consciousness	No	98	390.90 (326.55-477.22)	> 0.05
	Yes	16	408.05 (309.49-480.24)	
Vertigo	No	73	397.89 (323.68-481.85)	> 0.05
	Yes	41	372.86 (326.53-468.29)	
Visual impairment. diplopia. etc.	No	102	384.30 (316.06-468.96)	***< 0.05***
	Yes	12	453.84 (381.32-628.57)	

* Mann Whitney U Test

**Table 4:** Analysis Table of Variables with Isthmin-1

Variables		*N*	Isthmin-1	*p*-value
Systemic Region	Anterior System	59	378.0 (306.67-465.43)	> 0.05^#^
	Posterior System	50	416.69 (346.11-490.17)	
	Anterior+Posterior System	5	325.88 (202.45-1022.16)	
Middle cerebral artery	No	61	401.45 (345.45-485.46)	> 0.05*
	Yes	53	374.97 (298.60-461.24)	
Posterior cerebellar artery	No	68	378.71 (312.31-463.34)	> 0.05*
	Yes	46	399.67 (343.34-502.99)	
Internal carotid artery	No	105	397.89 (326.57-479.96)	> 0.05*
	Yes	9	366.38 (283.72-401.84)	
Anterior cerebral artery	No	108	399.67 (328.62-479.96)	= 0.005*
	Yes	6	290.40 (233.63-336.24)	
Basilar artery	No	107	392.61 (325.88-471.46)	> 0.05*
	Yes	7	440.33 (352.52-603.74)	
Superior cerebellar artery	No	110	393.72 (326.34-479.96)	> 0.05*
	Yes	4	390.75 (249.13-463.38)	
Posterior inferior cerebellar artery	No	107	390.39 (325.88-469.04)	> 0.05*
	Yes	7	471.46 (378.30-596.93)	
Lacunar	No	84	378.86 (327.07-468.61)	> 0.05*
	Yes	30	426.57 (279.19-489.25)	
Multiple vessels	No	106	393.72 (326.57-472.67)	> 0.05*
	Yes	8	353.09 (234.78-1388.05)	

^#^ Kruskal-Wallis Test, *Mann Whitney U Test

No significant differences in Isthmin-1 levels were observed with respect to mechanical thrombectomy status, Bamford classification, 90-day mortality, or 90-day functional outcome assessed by mRS (Table 5).

**Table 5:** Analysis Table of Variables with Isthmin-1

Variables		*N*	Isthmin-1	*p*-value
BAMFORD classification	PACI	45	378.0 (298.08-437.89)	> 0.05^#^
	POCI	46	393.54 (339.44-509.21)	
	LACI	23	418.53 (346.78-479.96)	
90-day modified Rankin scale	0	13	357.48 (281.19–472.70)	> 0.05^#^
	1	41	389.19 (326.53-468.38)	
	2	19	392.61 (310.43-497.17)	
	3	14	443.53 (341.14–488.70)	
	4	12	362.10 (287.94-741.02)	
	5	6	384.52 (311.64-1442.52)	
	6	9	417.66 (373.91-580.55)	
Mechanical thrombectomy	No	104	395.94 (326.03-479.05)	> 0.05*
	Yes	10	378.71 (347.96-529.57)	
90-day mortality	No	105	392.61 (323.68-478.13)	> 0.05*
	Yes	9	417.66 (373.91-580.55)	

^#^ Kruskal-Wallis Test, Mann Whitney U Test, PACİ; Partial anterior circulation infarction, POCİ; Posterior circulation infarction, LACİ; Lacunar infarction

Spearman correlation analysis showed a weak but statistically significant positive correlation between Isthmin-1 levels and ASPECTS score (*r* = 0.252, *p* = 0.007). No significant correlations were found between Isthmin-1 levels and age, symptom onset time, admission NIHSS score, or 90-day mRS score (Table 6).

**Table 6:** Correlation analysis of Isthmin-1 with variables

Variables		Age	Duration	On-admission NIHSS	ASPECT	90-day mRs
Isthmin-1	r	0.012	-0.019	-0.028	***0.252***	0.096
	p	0.900	0.841	0.765	***0.007***	0.309

mRS; Modified Rankin Scale, r; Spearman correlation coefficient

## Discussion

In this study, we investigated the diagnostic and prognostic significance of serum Isthmin-1 levels in patients with acute ischemic stroke. Ischemic stroke remains one of the leading causes of mortality, long-term disability, and cognitive impairment worldwide, with a substantial proportion of survivors experiencing persistent neurological deficits [9]. Early identification of biomarkers reflecting disease severity and underlying pathophysiological processes may facilitate improved risk stratification and support clinical decision-making in the acute phase of stroke.

Data regarding Isthmin-1 in the context of ischemic stroke are extremely limited. To the best of our knowledge, no previous clinical study has specifically evaluated serum Isthmin-1 levels in patients with acute ischemic stroke. Therefore, the present study represents the first clinical investigation exploring the potential role of Isthmin-1 in ischemic stroke pathophysiology.

Our findings demonstrated that serum Isthmin-1 levels were significantly higher in patients with ischemic stroke compared with healthy controls. This elevation may be related to the regulatory roles of Isthmin-1 in inflammatory responses, endothelial function, oxidative stress, and cellular energy metabolism. These processes are central components of ischemic brain injury and secondary neuronal damage, suggesting that Isthmin-1 may be actively involved in the acute phase of ischemic stroke.

Experimental and translational studies provide mechanistic support for this association. Li et al. demonstrated that Isthmin-1 expression is markedly upregulated in alveolar epithelial cells under hypoxic conditions and identified hypoxia-inducible factor-1α (HIF-1α) as a key transcriptional regulator of Isthmin-1 expression [7]. Hypoxia plays a pivotal role in ischemic stroke, during which HIF-1α becomes stabilized and orchestrates the expression of numerous genes involved in angiogenesis, metabolism, and cell survival. If Isthmin-1 is indeed a downstream target of HIF-1α, its increased expression during cerebral hypoxia may reflect an endogenous response to ischemic stress. Within the central nervous system, excessive Isthmin-1 expression could potentially influence blood–brain barrier permeability, thereby contributing to edema formation, neuroinflammation, and neuronal injury.

Additional evidence supporting the vascular relevance of Isthmin-1 comes from studies in non-neurological diseases. Shariri et al. reported increased Isthmin-1 expression in patients with glomerular kidney disease and demonstrated its inhibitory effects on angiogenesis and vascular function, along with impaired podocyte viability and differentiation [10]. Given that post-stroke recovery relies heavily on vascular repair and angiogenesis, elevated Isthmin-1 levels may negatively affect reperfusion and tissue recovery by suppressing re-angiogenesis in ischemic brain regions.

Moreover, several studies have linked serum Isthmin-1 levels to metabolic dysfunction, further underscoring its systemic relevance. Lopez-Yus et al. found a positive correlation between serum Isthmin-1 levels and the subcutaneous-to-visceral fat ratio in individuals with obesity [11]. Lei et al. reported that the combination of Isthmin-1 with triglyceride and uric acid levels was among the strongest predictors of metabolic dysfunction–associated fatty liver disease and metabolic syndrome [12]. Jiang and colleagues found that Isthmin-1 suppresses hepatic steatosis by switching from lipogenesis to protein synthesis; adipose-specific Isthm-1 caused severe fatty liver in mice [13]. In a study by Li and colleagues, Isthmin-1, an insulin-like adipokine secreted by mature adipocytes, was found to increase glucose uptake in adipocytes and skeletal muscle and activate the AKT/mTOR pathway [14]. Elevated Isthmin-1 levels have also been associated with insulin resistance in patients with polycystic ovary syndrome and primary hypertension, suggesting a broader role in metabolic and vascular dysregulation [15]. Since metabolic syndrome and endothelial dysfunction are well-established risk factors for ischemic stroke, these findings provide indirect support for the observed elevation of Isthmin-1 in our stroke cohort. Deng and colleagues found that serum Isthmin-1 may serve as a potential biomarker for abnormal urinary Na+ excretion and insulin resistance in individuals with primary hypertension [16]. In a study conducted by Hu and colleagues, Isthmin-1 was found to reduce cardiac ischaemia/reperfusion injury via the cGMP PKG signalling pathway and to improve age-related cardiac dysfunction by increasing glycolysis and SIRT1 activity [17]. Nguyen and colleagues identified Isthmin-1 as an endogenous anti-inflammatory lung protein; they found that its deficiency in mice led to spontaneous chronic inflammation, emphysema, and a condition similar to COPD [18].

In our study, the median serum Isthmin-1 level was 393.72 ng/mL in the ischemic stroke group compared with 209.64 ng/mL in healthy controls, representing a statistically significant difference (*p* < 0.001). Given the paucity of reference data in cerebrovascular disease, this finding is particularly noteworthy. Furthermore, patients with anterior cerebral artery (ACA) territory infarctions exhibited significantly lower Isthmin-1 levels, whereas those with posterior circulation infarctions tended to have higher levels. Consistently, patients presenting with visual disturbances—symptoms commonly associated with posterior circulation strokes—also demonstrated significantly different Isthmin-1 levels. These observations suggest that Isthmin-1 expression may vary according to vascular territory involvement, potentially reflecting differences in regional hypoxia, collateral circulation, or endothelial responses.

From a clinical perspective, we observed a significant positive correlation between serum Isthmin-1 levels and ASPECT scores, indicating an association with the radiological extent of ischemic injury. Although patients with poorer functional outcomes (mRS 3–6) exhibited relatively higher Isthmin-1 levels, this difference did not reach statistical significance. This finding suggests that while Isthmin-1 may reflect ischemic burden and early tissue injury, its standalone prognostic value for long-term functional outcomes may be limited.

Overall, our findings suggest that Isthmin-1 is involved in the acute pathophysiological processes of ischemic stroke, particularly those related to hypoxia, endothelial dysfunction, and vascular integrity. The observed association between serum Isthmin-1 levels and ASPECTS score supports its potential role as a marker of radiological ischemic burden rather than as a standalone predictor of long-term functional outcome.

Importantly, although patients with poorer functional outcomes tended to have higher Isthmin-1 levels, this association did not reach statistical significance. This finding indicates that the prognostic utility of Isthmin-1 may be limited when evaluated in isolation and suggests that its clinical value may lie in combination with established clinical, radiological, or laboratory parameters. Future studies incorporating larger, multicenter cohorts, serial biomarker measurements, and multivariable predictive models are warranted to further clarify the diagnostic and prognostic relevance of Isthmin-1 in ischemic stroke.

### Limitations

Several limitations should be considered when interpreting the results of this study. First, the single-center design and relatively small sample size may limit the generalizability of our findings. Larger, multicenter studies are needed to validate these results across diverse populations.

Second, despite the prospective design, some data loss occurred due to incomplete follow-up or difficulties in contacting patients during the 90-day follow-up period. Although the 90-day mRS is widely accepted for outcome assessment in stroke studies, it may not fully capture long-term neurological recovery.

Third, there was a significant age difference between the patient and control groups, with the control group being younger. Although no statistically significant correlation was observed between age and serum Isthmin-1 levels in our cohort, age-related changes in vascular biology, inflammation, and metabolic regulation may still have influenced biomarker levels. Therefore, age may have acted as a potential confounding factor in the comparison between groups. The lack of an age- and sex-matched control group limits our ability to attribute the observed differences in Isthmin-1 levels exclusively to ischemic stroke pathology.

Future studies with age- and sex-matched control populations and multivariable analyses are warranted to better clarify the independent role of Isthmin-1 in ischemic stroke.

## Conclusion

This study provides clinical evidence that serum Isthmin-1 levels are elevated in patients with acute ischemic stroke compared with healthy individuals. The associations observed between Isthmin-1 levels, vascular territory involvement, and radiological severity suggest that this molecule may reflect underlying hypoxic and endothelial mechanisms during the acute phase of ischemic stroke. Although serum Isthmin-1 levels were not independently associated with long-term functional outcomes, our findings support its potential role as an adjunctive biomarker in the early clinical assessment of ischemic stroke. Further large-scale, multicenter studies are needed to validate these findings and to explore the integration of Isthmin-1 into multimarker risk stratification strategies.

## Supplementary Information

Below is the link to the electronic supplementary material.

Supplementary Material 1
