# Supplementary material for: Diagnostic and clinical associations of serum Isthmin-1 in acute ischemic stroke
Source: Ir J Med Sci. 2026 Apr 16;195(4):2407–15. doi: 10.1007/s11845-026-04383-2 (PMC13588826; doi:10.1007/s11845-026-04383-2)
Supplement: Supplementary file 1 [file 11845_2026_4383_MOESM1_ESM.pdf]

Authorship Form

Irish Journal of Medical Science

Springer

Manuscript ID Number: IJMS-D-26-00534

Article Title: (first few words) Diagnostic and Clinical Associations of Serum Lithium...

First Author: Hasan Hüseyin Sarkı

E-mail: hasansarki61@gmail.com

AUTHORSHIP

I, the undersigned author(s), certify that:

- **I have seen and approved the final version of the manuscript, and all subsequent versions;**
- **I have made substantial contributions to conception and design, or acquisition of data, or analysis and interpretation of data;**
- **I have drafted the article or revised it critically for important intellectual content.**
- **I agree to be accountable for all aspects of the work in ensuring that questions related to the accuracy or integrity of any part of the work are appropriately investigated and resolved.**

I accept public responsibility for it, and believe it represents valid work. As an author of this article, I certify that none of the material in the manuscript has been previously published, nor is included in any other manuscript. I certify that this manuscript is not under consideration for publication elsewhere, nor has it been submitted or accepted in another publication in any form. The rights or interest in the manuscript have not been assigned to any third party.

Moreover, should the editor of *Irish Journal of Medical Science* request the data upon which the manuscript is based, I shall produce it. I also certify that I have read and complied with the copyright information, as found on the journal home page website.

After submission of this agreement signed by all authors, changes of authorship or in the order of the authors listed will not be accepted by Springer.

☑ Author's signature

Hasan Hüseyin SARKI / 10.02.2026

Printed name & date

☐ Author's signature

Yeşim İşler / 10.02.2026

Printed name & date

☑ Author's signature

Melih Yüksel 10.02.2026

Printed name & date

☐ Halil Kaya 10.02.2026

Author's signature

Printed name & date

☒ Demet Yıldız

Author's signature 10.02.26

Printed name & date

☐ Umut OCAK 10.02.2026

Author's signature

Printed name & date

☑ Author's signature

Kağan Huysal - 10.02.2026

Printed name & date

☐ Author's signature

Muhammed Said Karaca - 10.02.2026

Printed name & date

☒ Mehmet Oğuzhan Ay / 10.02.2026

İsmail Ayan / 10.02.2026

Completed forms can be scanned and included as a pdf file during the online submission process as a supplemental file not for review, or submitted by fax to the editorial office: +91 44 42197763

ICMJE DISCLOSURE FORM

**Date:**	2/10/2026
**Your Name:**	Melih Yüksel
**Manuscript Title:**	**Diagnostic and Clinical Associations of Serum Isthmin-1 in Acute Ischemic Stroke Abstract**
**Manuscript Number (if known):**	Click or tap here to enter text.

In the interest of transparency, we ask you to disclose all relationships/activities/interests listed below that are related to the content of your manuscript. "Related" means any relation with for-profit or not-for-profit third parties whose interests may be affected by the content of the manuscript. Disclosure represents a commitment to transparency and does not necessarily indicate a bias. If you are in doubt about whether to list a relationship/activity/interest, it is preferable that you do so.

The author's relationships/activities/interests should be defined broadly. For example, if your manuscript pertains to the epidemiology of hypertension, you should declare all relationships with manufacturers of antihypertensive medication, even if that medication is not mentioned in the manuscript.

In item #1 below, report all support for the work reported in this manuscript without time limit. For all other items, the time frame for disclosure is the past 36 months.

		**Name all entities with whom you have this relationship or indicate none (add rows as needed)**	**Specifications/Comments (e.g., if payments were made to you or to your institution)**
**Time frame: Since the initial planning of the work**			
**1**	All support for the present manuscript (e.g., funding, provision of study materials, medical writing, article processing charges, etc.) **No time limit for this item.**	☒ None	
			Click the tab key to add additional rows.
**Time frame: past 36 months**			
**2**	Grants or contracts from any entity (if not indicated in item #1 above).	☒ **None**	
**3**	Royalties or licenses	☒ **None**	

		Name all entities with whom you have this relationship or indicate none (add rows as needed)	Specifications/Comments (e.g., if payments were made to you or to your institution)
**4**	Consulting fees	☒ **None**	
**5**	Payment or honoraria for lectures, presentations, speakers bureaus, manuscript writing or educational events	☒ **None**	
**6**	Payment for expert testimony	☒ **None**	
**7**	Support for attending meetings and/or travel	☒ **None**	
**8**	Patents planned, issued or pending	☒ **None**	
**9**	Participation on a Data Safety Monitoring Board or Advisory Board	☒ **None**	
**10**	Leadership or fiduciary role in other board, society, committee or advocacy group, paid or unpaid	☒ **None**	

		Name all entities with whom you have this relationship or indicate none (add rows as needed)	Specifications/Comments (e.g., if payments were made to you or to your institution)
**11**	Stock or stock options	☒ **None**	
**12**	Receipt of equipment, materials, drugs, medical writing, gifts or other services	☒ **None**	
**13**	Other financial or non-financial interests	☒ **None**	

Please place an "X" next to the following statement to indicate your agreement:

☒ I certify that I have answered every question and have not altered the wording of any of the questions on this form.
